# Perceptual Expectations of Object Stimuli Modulate Repetition Suppression in a Delayed Repetition Design

**DOI:** 10.1038/s41598-018-31091-4

**Published:** 2018-08-21

**Authors:** Lisa Kronbichler, Sarah Said-Yürekli, Martin Kronbichler

**Affiliations:** 1 Centre for Cognitive Neuroscience and Department of Psychology, University of Salzburg, Salzburg, Austria; 2 Neuroscience Institute, Christian-Doppler Medical Centre, Paracelsus Medical University, Salzburg, Austria

## Abstract

Several fMRI and EEG/MEG studies show that repetition suppression (RS) effects are stronger when a stimulus repetition is expected compared to when a stimulus repetition is less expected. To date, the prevalent way to assess the influence of expectations on RS is via immediate stimulus repetition designs, that is, no intervening stimuli appear between the initial and repeated presentation of a stimulus. Since there is evidence that repetition lag may alter RS effects in a qualitative manner, the current study investigated how perceptual expectations modify RS effects on object stimuli when repetition lag is relatively long. Region of interest analyses in the left occipital cortex revealed a similar activation pattern as identified in previous studies on immediate lag: RS effects were strongest when repetitions were expected compared to decreased RS effects when repetitions were less expected. Therefore, the current study expands previous research in two ways: First, we replicate prior studies showing that perceptual expectation effects can be observed in object-sensitive occipital areas. Second, the finding that expectation effects can be found even for several-minute lags proposes that Bayesian inference processes are a relatively robust component in visual stimulus processing.

## Introduction

The repeated exposure to a stimulus leads to diminished neural response, a phenomenon commonly termed repetition suppression (RS^1,2^). RS effects were shown in animal single-cell recordings^3–5^ and in numerous studies with human subjects^6–10^. A few years ago, a study showed that RS for faces was reduced when repetitions were less expected^11^. This finding led to an ongoing discussion if top-down factors like expectation influence RS. The influence of experimentally induced perceptual expectations (by using periods with more frequent and less frequent repetitions) on RS effects has been replicated several times in fMRI^12,13^, EEG^14^ and MEG^15^ studies and for different stimulus types and modalities^15–21^. In these studies (and in the current one), expectations are manipulated via manipulating the frequency of repetitions. This is done by using blocks in which the frequency (and therefore the probability of repetitions) are manipulated. In this way, repetition probability (RP) is assumed to trigger perceptual expectations of repetitions by varying repetition probability across blocks. In should be noted that RP is just one way to trigger and manipulate perceptual expectations.

Notably, some studies do not replicate an effect of RP. Single-cell recordings in monkey inferior temporal cortex did not show an influence of RP on RS^22^ and examinations in humans not always replicate effects of RP^23^. Furthermore, the effect of RP might be restricted to familiar stimuli^18^ or require attention to the stimuli^12^.

Recent examination point out that RS (a reduced response towards *repeated* stimuli) and expectation suppression (ES; a reduced response towards *expected* stimuli), result from two different processes^24–26^. These studies use a different design, in which the first stimulus of a stimulus pair indicates whether a repetition is likely to occur in the second^24,25^. ES and RS effects were found largely independent from each other. Within predictive coding models^27,28^, RS and ES reflect the implementation of two predictions: a local prediction of a stimulus repetition due to changes in synaptic weights and a hierarchically higher expectation of a specific RP^19^. Due to its low temporal resolution the BOLD response in immediate repetition designs might be prone to reflect a mixture of both effects^29^.

Most previous studies that investigated the effects of RP on RS used immediate stimulus repetition designs, with only a small time lag and no intervening trials between initial and repeated stimulus presentation^12,13,16,18^. To examine whether RP effects (as measured with fRMI) are a robust finding, an investigation with longer time lags and several intervening stimuli is necessary. Similar to immediate repetition designs, decreased neural activity has been found for repeated compared to new stimuli with after longer lags^30–32^. Although both long and short-term repetitions are primarily associated with decreased neural activity for repeated stimuli, a number of studies suggests that there are qualitative differences between immediate and long-term priming (for a review in the domain of face processing see Henson^33^).

Besides the general question whether effects of RP on RS are also evident beyond immediate repetition, there is a more theoretical reason to examine RP for longer lags: Immediate repetition designs allow the formation of exact expectations about forthcoming stimuli (*if* there is a repetition, it will be the exact same stimulus). By contrast, in long term repetition designs, the exact prediction about which stimulus will appear next is not possible. Here, the only expectation that can be formed is whether an increased or a decreased number of previously seen (e.g., repeated) stimuli are to be expected in a specific block (depending on the proportion of stimulus repetition in each block). If the modulation of RS by RP is solely due to exact expectations of a specific stimulus, we would not expect effects of RP. Alternatively, if less specific expectations are sufficient to influence RS effects, we would expect to find an influence of RP on RS effects as measured with fMRI.

Within the framework of predictive coding, one might expect such an influence: The brain does not merely signal whether a certain stimulus is expected or not (and whether this expectation was fulfilled) in the sense of a binary signal. Prediction errors in these models are continuously calculated during object perception. Errors are minimized by updating predictions from hierarchically higher regions which continuously update their predictions and try to explain away prediction errors by altering synaptic weights^34^. When a stimulus is encountered repeatedly, hierarchically higher regions are relatively faster in explaining away prediction errors. Accordingly, we might expect reduced prediction errors for repeated stimuli, even if an exact prediction of the stimulus is not possible.

Based on these assumptions, the present study examined whether fMRI RS is modulated by RP in a long-term repetition design with a lag of several minutes and numerous intervening stimuli. By now, effects of RP have exclusively been shown for immediate repetitions and a modulation of RS by expectations (manipulated via RP) for longer lags would extent the generality of this finding.

Participants performed a silent naming task on visually presented objects. After a several minutes break, they completed a second run with object images. Images were either taken from the first part (repeated) or were completely new. Stimuli were arranged in blocks with either a high RP (75% repetitions) or a low RP (25% repetitions). Our main analyses focused on the lateral occipital cortex as the region was regularly found to be object-sensitive^2,35,36^ and has commonly been targeted in fMRI assessments on RS^2,37^ and RP^16,23^. Since our previous study revealed that effects of RP on RS were only evident in the left LOC, we examined the left and right LOC separately. The goal of the present study was not to directly contrast different lags against each other (and therefore to compare short versus long lags), but to explore whether RP has any effect on RS in situations beyond the previously used immediate repetition design.

## Methods

### Participants

Thirty-six (24 female) undergraduate students from the University of Salzburg and adult citizens of Salzburg (age 17 to 41 years) participated in the present study. All participants had normal or corrected-to-normal vision and reported no history of neurological or psychiatric disease. They provided written informed consent and were remunerated for participation with structural images of their brains on CD and European Credit Transfer System credits. All methods conform to the Code of Ethics of the World Medical Association (Declaration of Helsinki) and were in accordance with the institutional guidelines of the University of Salzburg. The institutional guidelines of the University of Salzburg (Statutes of the University of Salzburg - see https://online.uni-salzburg.at/plus_online/wbMitteilungsblaetter.display?pNr=98160) state in § 163 (1) that ethical approval is necessary for research on human subjects if it affects the physical or psychological integrity, the right for privacy or other important rights or interests of the subjects or their dependents. In § 163 (2) it is stated that it is the responsibility of the PI to decide, whether (1) applies to a study or not. Therefore, we did not seek ethical approval for this study. Since it was non-invasive and performed on healthy adult volunteers who gave their informed consent to participate, (1) did not apply. Data was processed in anonymized/deidentified form. Upon arrival at the lab, participants were assigned a subject ID (v001, v002, etc.) which was used throughout the study.

### Stimuli and design

**Figure 1:**
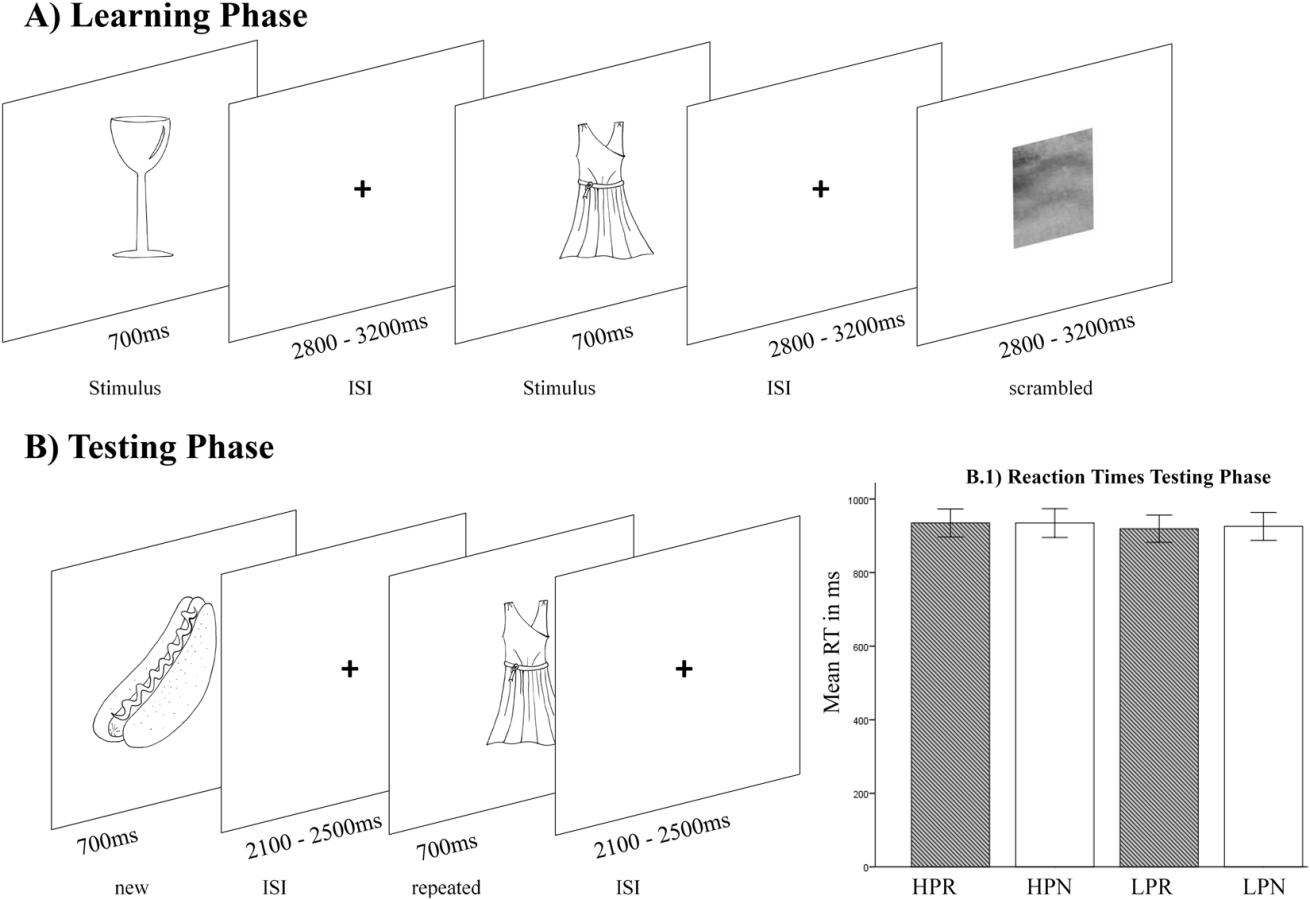
Example line drawings as similarly used in both phases (**A**): learning phase; (**B**) testing phase) of our experiment. Images for this illustration purpose were drawn by the first author. B1 depicts participants’ reaction times on the scanner task. *Abbreviations*: ISI = Inter-stimulus-Interval; HP = high probability; LP = low probability; N = new stimulus; R = repeated stimulus.

The experimental setup included a learning and a testing phase. Images presented in both phases were 300 × 300 px monochrome object line-drawings from a standardized corpus^38^ (example images are illustrated in Fig. 1). Additional pictures downloaded from the public domain of the World Wide Web were matched in size and luminance to the pictures of the corpus. Pictures were presented centrally on a white background through the scanner bore onto a mirror (at a distance of approximately 80 cm), which reflected the image to the participant.

In the learning phase, participants observed 280 object line-drawing (each showing a unique object), ten percent null events (depicting a fixation cross) and 40 scrambled images. Each stimulus remained on screen for 700 ms and was followed by the next stimulus after a jittered inter-stimulus interval (mean: 3000 ms; range: 2800 ms–3200 ms). Null events and scrambled images were randomly distributed across stimuli (see Fig. 1). Participants were asked to silently name the objects and press a button when finished. They were not aware that the images of the learning phase would be relevant in the testing phase.

The testing phase was preceded by a 5 minutes break, during which the MPRAGE structural scans were acquired. To prevent participants from reflecting on previously observed images, they were shown a short movie showing Hubble’s view of the universe (the movie neither included relevant objects nor speech).

In the subsequent testing phase, participants observed 560 stimuli. Half of the images were taken from the testing phase (old) and the other half consisted of completely new images (new). Object line-drawings were presented in 14 blocks, separated by jittered breaks where a fixation cross was shown (mean inter-block interval: 14000 ms; range: 10000 ms–18000 ms). Stimulus blocks alternated between high-probability blocks and low-probability blocks. Each block consisted of 40 stimuli. Stimuli remained on screen for 700 ms and were followed by a jittered inter-stimulus interval (mean: 2300 ms; range: 2100–2500 ms). In a high-probability block, 75% of stimuli (n = 30) were taken from the learning phase and 25% of stimuli (n = 10) were new. Therefore, the probability of a stimulus being repeated was relatively high. In a low-probability block, 25% of stimuli (n = 10) were taken from the learning phase and 75% of stimuli (n = 30) were new. Therefore, the probability of a stimulus being repeated was relatively low. The first three stimuli of a low-probability block were always new stimuli and the first three stimuli of a high-probability block were always old. To warrant participants’ attention during this phase^12^, they should indicate via button press (yes or no) whether the currently depicted stimulus would fit into an imaginary shoebox. Half of the images fitted into a shoebox and the other half did not. Additionally, shoebox fitting was kept equally across blocks.

### Image acquisition and Data analysis

Functional imaging data were acquired with a Siemens Magnetom Trio 3 Tesla scanner (Siemens AG, Erlangen, Germany) equipped with a 12-channel head-coil. Functional images sensitive to blood oxygen level dependent (BOLD) contrast were acquired with a T2* weighted gradient echo EPI sequence (TR 2250 ms, TE 30 ms, matrix 64 × 64 mm, FOV 192 mm, flip angle 70°). Thirty-six slices with a slice thickness of 3 mm and a slice gap of 0.3 mm were acquired within the TR. Scanning proceeded in 2 sessions with 469 and 833 scans, respectively. Six dummy scans were acquired at the beginning of each functional run before stimulus presentation started. Additionally, a gradient echo field map (TR 488 ms, TE 1 = 4.49 ms, TE 2 = 6.95 ms) and a high resolution (1 × 1 × 1.2 mm) structural scan with a T1 weighted MPRAGE sequence were acquired from each participant.

For preprocessing and statistical analysis, SPM12 software (http://www.fil.ion.ucl.ac.uk/spm/), running in a MATLAB R2013a environment (Mathworks Inc., Natick MA, USA), and additional functions from AFNI (https://afni.nimh.nih.gov/) were used. The exact processing steps are provided in the Supplementary Material.

Rescaled contrast images (see Supplementary Material) were used in second level analyses for a 2 (stimulus type: repeated and new) × 2 (condition: high-probability and low-probability) ANOVA. All results from whole brain analysis are reported at a voxel-level threshold of *p* < 0.001 (uncorrected) with a FWE cluster-level correction of *p* < 0.05. The datasets generated and analysed during the current study are available from the corresponding author on reasonable request.

### ROI Analyses

**Figure 2:**
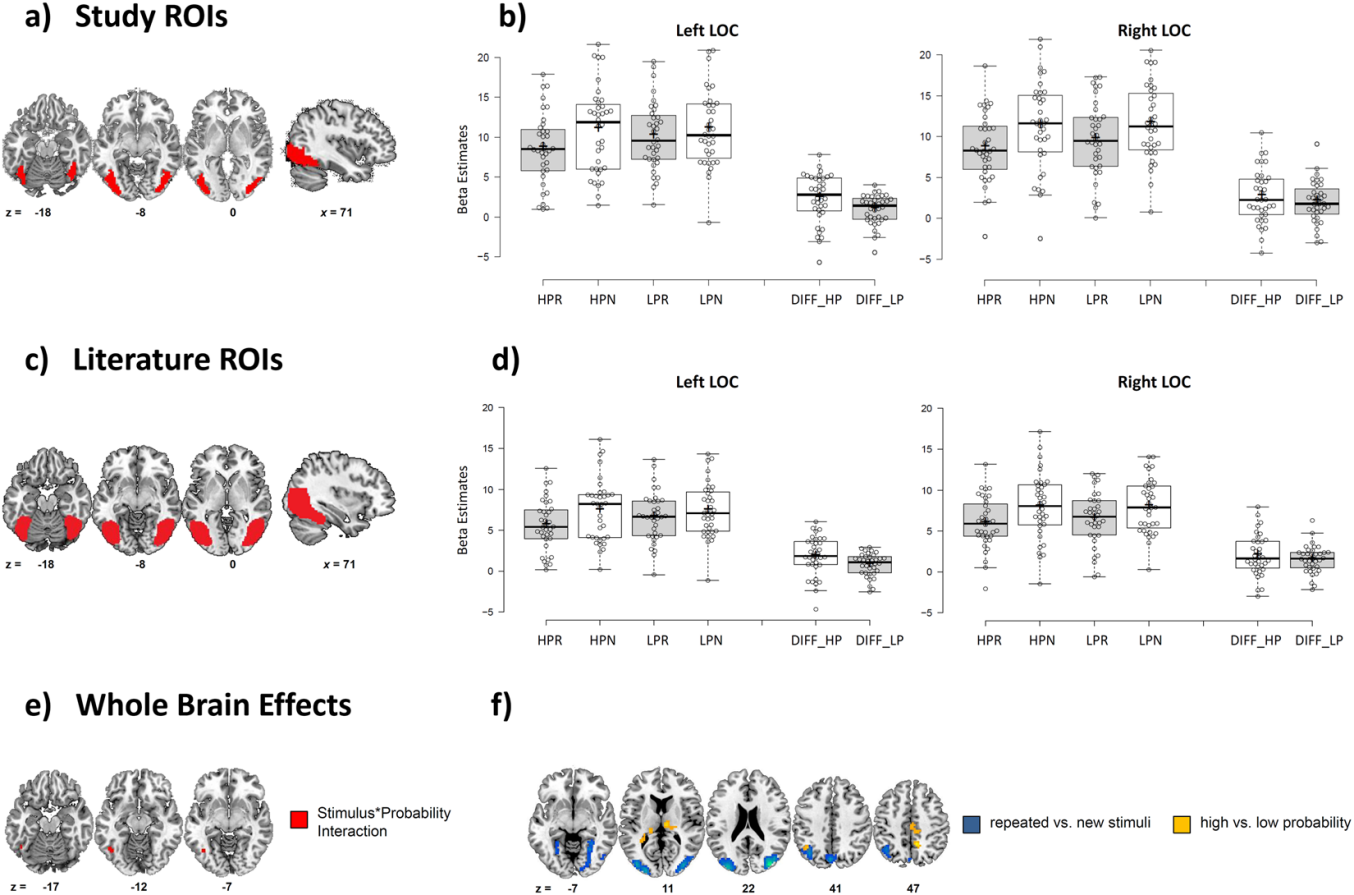
(**a)** Red cluster illustrate left and right LOC group ROI defined based on the *object drawings* > *scrambled stimuli t*-contrasts (*p* < 0.05 [FWE corrected]) of the image learning phase. (**b)** Tukey Box-plots depict beta estimates extracted from left and right lateral occipital ROI. Bold horizontal lines indicate the group median, bold crosses show the group mean. End of whiskers indicate the first and third quartile. Abbreviations: HP = High Probability Context; LP = Low Probability Context; R = Repeated Stimulus; N = New Stimulus; DIFF_HP shows differential scores calculated by subtracting mean subject beta estimates for repeated stimuli from mean subject beta estimates for new stimuli in the high probability condition. DIFF_LP shows differential scores calculated by subtracting mean subject beta estimates for repeated stimuli from mean subject beta estimates for new stimuli in the low probability context. (**c)** Red cluster illustrate left and right LOC group ROI taken from Julian *et al*.^40^. (**d)** Tukey Box-plots depict beta estimates extracted from left and right lateral occipital ROI taken from Julian *et al*.^40^. For description see in (**b**). (**e)** Activation cluster revealed by the whole brain analyses. The significant stimulus-by-probability interaction cluster is depicted in red (voxel-level correction *p* < 0.001 [uncorrected] and cluster level correction *p* < 0.05 [FDR]). (**f)** Activation clusters revealed by the whole brain analyses. Significant repeated vs. new stimuli contrast clusters are depicted in blue. Yellow marks clusters where the main effect of probability (high vs. low probability) was significant. All clusters were extracted at a voxel-level correction of p < 0.001 (uncorrected) and a cluster level correction of p < 0.05 (FWE).

Left and right lateral occipital complex (LOC) were defined based on voxel-based group analyses of the learning phase by using the *object drawings* > *scrambled stimuli t*-contrasts. To define the LOC, we applied a voxel-level threshold of *p* < 0.05 (FWE corrected) on the group contrast. This definition led to the following locations in MNI space and sizes for the ROIs: left LOC *-45 -73 -8* (388 voxels) and right LOC *39 -85 -8* (289 voxels). The exact location and extent of our bilateral LOC cluster is illustrated in Fig. 2a.

Box-plots were designed with an R-based web tool described in Spitzer *et al*.^39^. We also used two alternative methods to define ROIs for the left and right LOC. In the first, we used group ROIs gained from Julian *et al*.^40^. These ROI masks are freely available at (http://web.mit.edu/bcs/nklab/GSS.shtml). Extent and location of these alternative group ROIs can be seen in Fig. 2c. In the second, we individually defined left and right LOC cluster for each participant, based on the *object drawings* > *scrambled stimuli t*-contrasts (*p* < 0.001, uncorrected) of the learning phase. Individual coordinates and cluster extent are listed in Supplementary Table 1.

## Results

### Behavioral Data

RT was examined with a 2 × 2 repeated measures ANOVA with the factors probability (high versus low) and stimulus (repeated versus new). Reaction times did not vary with probability context or stimulus type, since no significant main effects of probability (*F*(1, 35) = 2.147, *p* = 0.152) and stimulus (*F*(1, 35) = 0.201, *p* = 0.657) type were observed. Similarly, the stimulus-by-probability interaction did not reach significance level (*F*(1, 35) = 0.192, *p* = 0.664). Results are depicted in Fig. 1.

### Left LOC

Left LOC RS effects were modulated by RP as evident by a significant stimulus-by-probability interaction (*F*(1, 35) = 5.569, *p* = 0.024). This interaction was characterized by strong RS effects in the high-probability condition (21% decrease in BOLD response for repeated stimuli) and minimal RS in the low-probability condition (8% decrease in BOLD response). For difference scores see Fig. 2b. Repeated stimuli showed decreased BOLD response compared to new stimuli (*F*(1, 35) = 29.035, *p* < 0.001). No main effect of probability type could be observed (*F*(1, 35) = 1.97, *p* = 0.169). To explore whether this interactions was mainly due to differences between repeated trials in the two RP contexts (and not due to differences between novel trials between the high and the low RP context) we calculated pair-wise comparisons. These comparisons revealed a robust and statistically significant difference between repeated stimuli in the high RP compared to the low RP blocks (*t*(35) = 2.62, *p* = 0.013). No significant difference was found between new images in high versus low RP trials (*t*(35) = 0.15, *p* = 0.89).

### Right LOC

RS effects were similar in high and low probability conditions with 23.5% and 17% BOLD decrease for repeated stimuli, respectively (see Fig. 2b). Repeated stimuli showed decreased neural response compared to new stimuli (*F*(1, 35) = 69.68, *p* < 0.001). No stimulus-by-probability interaction and no main effect of probability context were found (*F*(1, 35) < 1.06, *p* > 0.3).

### Robustness of ROI results

To examine the robustness of our results we re-ran the 2 × 2 ANOVA in two additional analyses with different LOC ROI definition methods.

#### Literature-based ROI analysis

Left LOC: RS effects were modulated by RP as shown by a significant stimulus-by-probability interaction (*F*(1, 35) = 5.614, *p* = 0.023). RS effects were evident in the high-probability condition (24% decrease in BOLD response for repeated stimuli) and decreased RS effects were found in the low-probability condition (11% decrease in BOLD response). Repeated stimuli revealed decreased BOLD response compared to new stimuli (*F*(1, 35) = 31.194, *p* < 0.001). No main effect of probability type was observed (*F*(1, 35) = 1.48, *p* = 0.232). ROI clusters and results are illustrated in Fig. 2c,d.

Right LOC: showed similar RS effects in high and low probability conditions with 25% and 20% BOLD decrease for repeated stimuli, respectively. Repeated stimuli showed decreased BOLD response compared to new stimuli (*F*(1, 35) = 60.98, *p* < 0.001). No stimulus-by-probability interaction and no main effect of RP was found (*F*(1, 35) < 0.83, *p* > 0.36).

#### Individually defined ROIs

Left LOC: RS effects were modulated by RP as shown by a significant stimulus-by-probability interaction (*F*(1, 35) = 5.878, *p* = 0.021). Pronounced RS effects could be observed in the high-probability condition (22% decrease in BOLD response for repeated stimuli) and decreased RS effects in the low-probability condition (8% decrease in BOLD response). Repeated stimuli revealed decreased BOLD response compared to new stimuli (*F*(1, 35) = 30.17, *p* < 0.001). No main effect of probability type was observed (*F*(1, 35) = 1.959, *p* = 0.170).

Right LOC: showed comparable RS effects in high and low probability conditions (22% and 17% BOLD decrease for repeated stimuli, respectively). Repeated stimuli showed decreased neural response compared to new stimuli (*F*(1, 35) = 61.39, *p* < 0.001). No stimulus-by-probability interaction and no main effect of probability context were found (*F*(1, 35) < 0.47, *p* > 0.49).

### Whole Brain Analysis

All results from whole brain analysis are reported at a voxel-level threshold of *p* < 0.001 (uncorrected) with a FWE cluster-level correction of *p* < 0.05.

Repeated stimuli revealed decreased BOLD response in widespread left and right middle occipital clusters, reaching from inferior to superior occipital gyrus. Decreased response for repeated stimuli was also found in a right fusiform and a left lingual gyrus cluster. The reverse pattern (more activation for repeated compared to new stimuli) was shown in a left precuneus region and the left angular gyrus. Findings are illustrated in Fig. 2e,f) and peak coordinates are listed in Table 1.

**Table 1:** Peak Coordinates and Cluster Sizes of the Voxel-Based Whole Brain Analysis.

Region	MNI coordinates			Volume	*F*	
	x	y	z	(voxels)		
*Repeated vs*. *New Stimuli*						
R middle occipital gyrus	33	−82	22	360	48.71	*A* > *R*
L middle occipital gyrus	−33	−85	19	338	42.93	*A* > *R*
R fusiform gyrus	27	−46	−11	229	33.28	*A* > *R*
Precuneus	−3	−73	40	106	29.94	*R* > *A*
L angular gyrus	−36	−64	40	97	28.02	*R* > *A*
L lingual gyrus	−24	−46	−11	108	26.45	*A* > *R*
*High vs*. *Low Probability Context*						
R precuneus	9	−49	46	69	41.89	*L* > *H*
R Thalamus	12	−13	4	102	35.73	*L* > *H*
L angular gyrus	−39	−58	40	33	26.74	*H* > *L*
L Thalamus	−18	−25	13	56	26.40	*L* > *H*

Note: Data were extracted at a voxel-level threshold of p < 0.001 (uncorrected) and a cluster level threshold (FWE) of p < 0.05.

Increased neural activation for the low compared to the high RP condition was found in bilateral thalamus as well as in a cluster in the right precuneus. Increased response for the high-probability context was shown in the left angular gyrus.

The stimulus-by-probability interaction did not exceed the cluster-level correction of *p* < 0.05 (FWE) in any cortical region. If cluster level correction was set to *p* < 0.05 (FDR), however, a small cluster (19 voxels) in the left fusiform gyrus emerged, which revealed a similar activation pattern as the ROI analysis in left lateral occipital.

Additional fMRI data analyses including the factor *hemisphere* are provided in the supplementary material.

## Discussion

Several recent studies found that repetition probability (RP) influences repetition suppression (RS) effects in the human brain as measured with fMRI and neurophysiological measures^11,14–16,18,41^. Under some circumstances however, such RP effects could not be observed^12,18^. All of these previous studies used immediate repetition designs, where repetitions occurred without intervening stimuli and with a short time lag between initial and repeated stimulus.

In the current study, we found, for the first time, a modulation of RS in the BOLD response by RP in a delayed repetition paradigm. This modulation of RS by RP was evident in a region-of-interest analysis for the left LOC. This finding was robust irrespective of the exact definition of the LOC: Findings remained the same when ROIs were individually defined or when literature based ROIs^40^ were used. The effect was also found in a whole-brain voxel-based analysis using an FDR-corrected cluster level threshold in a region in the left fusiform occipito-temporal cortex (closely corresponding to the LOC). In line with our previous study^16^, this modulation of RS by RP was not found in the right LOC, although an analysis with hemisphere as additional factor did not reveal a reliable interaction with hemisphere.

Please note that we refrain from providing a direct statistical comparison of our present result with that of our previous study (which used an immediate repetition design) due to a number of factors (e.g. differences in task, scan parameter, participants). However, a purely numerical and descriptive comparison reveals a close correspondence of effects of RP on RS in the present study and previous studies. RS effects in the left LOC ROI (−21% in high RP and −8% in low RP) resemble RS effects identified for immediate lag during object (−21% in high RP and +1% in low RP in Mayrhauser *et al*.^16^) and face processing (−22% in high RP and −9% in low RP in Summerfield *et al*.^11^). Future studies are needed to examine potential differences between RP effects on immediate and short-term and more long-term forms of RS.

Nonetheless, the present finding of an influence of RP on RS clearly demonstrates that such effects are not restricted to immediate repetitions. The present demonstration of reduced RS for less frequent (and less expected) repetitions also for delayed repetitions is important because a number of previous studies suggest quantitative and qualitative differences between short-term and long-term RS^33,42^. Our finding also demonstrates that an exact expectation about which specific stimulus will be repeated might not be necessary for an influence on RS. In contrast to immediate repetition designs, where the brain is able to generate specific expectations about the forthcoming stimulus, such a specific expectation could not be formed in our design.

As stated in the Introduction, effects of RP and expectations on RS have mostly been interpreted in terms of predictive coding (PC), where perception is cast as bayesian inference. Such inference dependents on recursive message passing between different levels of the cortical hierarchy with a central role of prediction errors and the influence of precision on prediction errors^19,43,44^.

In the field of RS, diverse variants of PC models have been proposed. Whereas some highlight a strong interplay between perceptual expectations and factors such as RP^45^, other PC models have proposed a relative independence between expectation suppression and RS^26^. This assumption is based on (a) the observed independence between expectation and repetition effects in designs when these were independently modulated by cues signalling trial-wise the probability of repetitions^25,46,47^ and (b) the finding that ES and RS effects are found at different time-points in neurophysiological measures with higher temporal resolution^24,48^. It is important to note that the independence between expectation and repetition effects was not found in all studies that independently manipulated the two effects^26,46^. Furthermore, in the MEG study of Todorovic *et al*.^24^, there was a trend towards an interaction between repetition and expectation effects in some mid-latency time-points. The present study cannot resolve the discussion on whether and to which extent RS can be explained by PC, but provides evidence for effects of RP on RS in the brain for long-term repetitions, a finding that models of RS (including those based on PC) will need to explain.

One might object that the present findings could also be explained by effects of attention^12,49^ and/or novelty intermingled with “pure” RS effects. Although we cannot fully rule out such explanations, we consider this possibility rather unlikely. On this account, one would not only expect that repeated stimuli lead to higher BOLD activity in low RP blocks compared to repetitions in high RP blocks (as the former are less expected than the latter and therefore associated with more novelty and attentional demands), but also that novel stimuli in high RP blocks lead to higher BOLD activity than novel stimuli in low RP blocks (again the former should be more surprising than the latter ones). This expectation was not reflected in our data for the left LOC: There was a significant difference between repeated stimuli in the high and low RP blocks, but no significant difference between novel stimuli in the two RP contexts. This statistical analysis has to be interpreted with caution, since our design was not ideal with respect to the statistical efficiency of these contrasts^50^. It nevertheless suggests that the presently found interaction was primarily caused by differences between the repeated stimuli and not between the novel stimuli. This pattern argues against an interpretation based on differences in novelty signals and/or stimulus-triggered attention.

Furthermore, we did not find any significant effects of RP in our behavioral data, whereas one might expect such effects if our stimulus types would trigger different amounts of attention. A recent EEG study^49^ found effects of RP on RS even during unconscious masked priming which is hard to reconcile with the idea that effects of RP primarily reflect effects of attention and or novelty detection. Also, novelty signals in the brain have primarily been associated with activity in the medial temporal lobe, especially the hippocampus, and not the LOC. Nevertheless, it is possible that RS aids in the process of detecting novel stimuli since this system provides a mechanism not only for the detection of repetitions but also for novelty detection^2^, because neural response towards new stimuli differs from repeated stimuli. Despite these considerations, it should be noted that we cannot completely rule out that the interaction between RS and RP might reflect a novelty signal. Future studies will be needed to gain further definite evidence on this question.

Another question for future research is to which degree subjects acquire conscious awareness of repetition manipulations and whether such awareness is needed for RP effects. None of our subjects reported any awareness of the RP manipulation after the experiment when asked if they had noticed something about the block structure. This is in line with other studies that used a RP manipulation^18^ and with a recent EEG study that found effects on RP on RS in an unconscious masked priming paradigm^49^. A more structured and in-depth debriefing and more advanced measurement methods^51^ might be needed to explore this issue in more detail.

A further limitation of the current study is that we cannot clarify why RP effects on RS are regularly identified in human BOLD fMRI and EEG^11,14,16^ studies but not in single-cell recordings in inferior temporal monkey cortex^52^. A common explanation was that macaques were less attentive (passive fixation task) compared to human participants (stimulus detection task) during the task. However, the recent finding that RP effects also occur unconsciously^49^ challenges this assumption. It should be the focus of future studies to systematically examine under which circumstances single-cell recordings and EEG or fMRI assessments do or do not reveal RP effects and whether such differences can be explained by the specific method or species examined.

Taken together, while previous studies focused on RP effects during immediate lag, the present study is the first to show such an effect for long lags. In line with previous studies of expectation effects and RP, we interpret these RP effects as top-down effects on RS. It should be noted that future studies will have to clarify whether RP effects really reflect top-down effects by showing that such effects originate in hierarchically higher brain regions and directly influence RS in hierarchically lower areas. Clever experimental designs and connectivity methods will be needed for such demonstrations. At the moment, the interpretation of RP effects as top-down effects remains somewhat speculative. In summary, our findings provide first evidence of potential top-down effects of RP during long-term repetition suppression and underline the robustness of RP effects on RS even when many trials intervene.

## Electronic supplementary material

Supplementary Data
