# Supplementary material for: Perceptual Expectations of Object Stimuli Modulate Repetition Suppression in a Delayed Repetition Design
Source: Sci Rep. 2018 Aug 21;8:12526. doi: 10.1038/s41598-018-31091-4 (PMC6104074; doi:10.1038/s41598-018-31091-4)
Supplement: Supplementary file 1 — Supplementary Data [file 41598_2018_31091_MOESM1_ESM.doc]

Supplementary Material

Perceptual Expectations of Object Stimuli Modulate Repetition Suppression in a Delayed Repetition Design

Lisa Kronbichler^b*^, Sarah Said-Yürekli^a,b^ & Martin Kronbichler^a,b^

^a^ Centre for Cognitive Neuroscience and Department of Psychology, University of Salzburg, Salzburg, Austria

^b^ Neuroscience Institute, Christian-Doppler Medical Centre, Paracelsus Medical University, Salzburg, Austria

*Methods*

*fMRI data acquisition*

Functional images were realigned, de-spiked (with the AFNI 3ddespike function), unwarped and corrected for geometric distortions using the fieldmap of each participant and slice time corrected. The high resolution structural T1-weighted image of each participant was processed and normalized with the CAT12 toolbox (http://dbm.neuro.uni-jena.de/cat) using default settings, each structural image was segmented into gray matter, white matter and CSF and denoised, then each image was warped into MNI space by registering it to the DARTEL template provided by the CAT12 toolbox via the high-dimensional DARTEL (Ashburner, 2007) registration algorithm. Based on these steps, a skull stripped version of each image in native space was created. To normalize functional images into MNI space, the functional images were coregistered to the skull stripped structural image and the parameters from the DARTEL registration were used to warp the functional images, which were resampled to 3 x 3 x 3 mm voxels and smoothed with a 6 mm FWHM Gaussian kernel.

Statistical analysis was performed with a GLM two staged mixed effects model. In the subject-specific first level model, each of the four critical condition (HPA, HPR,, LPA; LPR) was modelled by convolving stick functions at its onsets with SPM12’s canonical hemodynamic response function (target trials and start and end messages were modelled in the same way as separate events of no interest, the model also included the six motion parameters and six noise regressors, reflecting physiological noise components obtained from FIACH (Tierney et al., 2016) as regressors of no interest). Parameter estimates for each condition were calculated via these first level general linear models (GLM), using a temporal high-pass filter (cutoff 128 sec) to remove low-frequency drifts and modeling temporal autocorrelation across scans with an AR (1) process (Friston et al., 2002).

For the voxel-based group analyses, contrasts for the effects of interest were calculated at the first level and rescaled to increase statistical sensitivity and decrease inter-individual variability by the Vascular auto-rescaling of fMRI (VasA fMRI) technique (Kazan et al., 2016). The effects of interest were the main effect of repetition suppression (HPA + LPA minus HPR and LPR), the main effect of RP (HPA+HPR minus LPA+LPR) and the interaction (HPA minus HPR minus LPA plus LPR). For the ROI-based analysis contrast estimates for each of the four conditions of interest (HPA,HPR; LPA, LPR) were extracted for the different ROIs and used in 2x2 repeated measures ANOVAs for each ROI.

*Results*

*3.1 Behavioral Results*

To examine whether contextual effects were driven by attentional differences (for details on the topic see Larsson and Smith, 2012), reaction time was compared between conditions. No significant difference between High Probability Context (M=934.87, SD=232.95 ) and Low Probability Context (M=922.47, SD=226.21) could be observed (*t* = 1.46, *p* = .125). Since many participants reported that the stimuli were ambiguous with respect to the task (whether it fitted in a shoebox), we refrained from examining correct and incorrect responses since the task was merely used to keep participants attentive.

*ROI analyses: hemisphere*

To assess whether repetition probability context modulated RS effects differently in the left and right LOC ROIs, we conducted an additional 2x2x2 ANOVA with the factors hemisphere (left, right), probability (high, low) and stimulus (repeated, new). The critical 3-way interaction (hemisphere-by-probability-by-stimulus was not significant (*F*(1, 35) = 2.39, *p* = 0.193) and there was no significant main effect of hemisphere (*F*(1, 35) = 0.11, *p* = 0.742) However, there was a significant hemisphere-by-stimulus interaction (*F*(1, 35) = 11.064, *p* = .002).
